# Supplementing maternal diet with milk oligosaccharides and probiotics helps develop the immune system and intestinal flora of offsprings

**DOI:** 10.1002/fsn3.3579

**Published:** 2023-08-16

**Authors:** Qinggang Xie, Dongying Cui, Qinchao Zhu, Xuewen Qin, Daxi Ren, Xiaoxi Xu

**Affiliations:** ^1^ College of Food Science Northeast Agricultural University Harbin China; ^2^ Heilongjiang Feihe Dairy Co., Ltd. Beijing China; ^3^ Institute of Dairy Science, College of Animal Sciences Zhejiang University Hangzhou China

**Keywords:** immune system, intestinal flora, milk oligosaccharides, probiotics

## Abstract

Intestinal flora is very important for improving the development of the immune system in newborns. Maternal diet during pregnancy and lactation is one of the key factors affecting the growth and development of offspring. The objective of the present study was to examine whether supplementation of maternal diet with milk oligosaccharides and *Bifidobacterium* could influence the development of the intestinal flora and immune system of neonatal mice. In total, 30 pregnant Institute of Cancer Research (ICR) mice were randomly divided into six groups: a control group (basal diet) and five intervention groups (basal diet supplemented with different doses of 2′‐fucosyllactose [2′‐FL] and *Bifidobacterium Bb12*) during the pregnancy period. All female mice were monitored for physical health during gavage. After delivery, the number of mice in each litter, any deformity, and the development of the offspring were recorded. The spleen, blood, and fecal samples of six groups of 10–12 day‐old offspring were collected. The results demonstrated that maternal milk oligosaccharides and probiotics conferred protective effects against lipopolysaccharide (LPS)‐induced immunosuppression in mice offspring by significantly enhancing the immune organ indexes, splenocyte proliferation, immunoglobulin (immunoglobulin G, A, M) production as well as improving the macrophage phagocytosis (*p* < .05). The abundance of *Lactobacilli* and *Bifidobacteria* in the feces of offspring mice in the intervention groups was significantly higher than that of the offspring mice in the control group (*p* < .05). These findings suggest that the combination of 2′‐FL and *Bifidobacterium Bb12* displayed synergistic interactions between the two components that could promote the development of the immune system of the offsprings and improve their microbiota through maternal ingestion.

## Graphical abstract

Intestinal flora is very important for improving the development of the immune system in newborns. Maternal diet during pregnancy and lactation is one of the key factors affecting the growth and development of offspring. This study suggests that supplementation of maternal diet with milk oligosaccharides and *Bifidobacterium* could influence the development of the intestinal flora and immune system of neonatal mice.

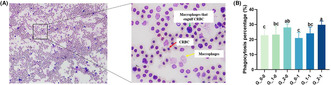

## 1 INTRODUCTION

The gut microbiota not only participates in various metabolic processes in the human body but also plays a critical role in host immune responses (Yang et al., 2020). Particularly, during early life, the microbiome profoundly influences the development and maturation of the gastrointestinal tract mucosa, which may affect health in later life (Thum et al., 2012). The first bacteria in the gastrointestinal tract of newborns originate from the maternal birth canal and breast milk and the environment (Wampach et al., 2018; Wang, Hu, et al., 2021; Wang, Ze, et al., 2021). Following parturition, bacteria rapidly colonize the relatively sterile gastrointestinal tract of newborns, marking the beginning of the highly complex process of microbiome establishment (Bäckhed et al., 2015; Ray, 2016). The maternal gut and vaginal microbiota and breast milk composition are influenced by the mother's diet (Kominiarek & Rajan, 2016; Thum et al., 2012). The supplementation of probiotics and prebiotics reportedly improves maternal microbiome composition, consequently inducing the colonization of beneficial bacteria in the infant gut (Schultz et al., 2004). Gut microbiota facilitates folate production, reduces allergic diseases, enhances immune response to vaccination, and synthesizes essential vitamins and other molecules (Miao et al., 2018; Tamburini et al., 2016). The early establishment and colonization of gut microbiota has several advantages, such as promoting the development and maturation of gut immunity in addition to maintaining the host barrier function (Kabat et al., 2014). Thus, the transition between gestation and lactation periods can serve as a window of opportunity for maternal dietary intervention, thereby modulating the intestinal health of offsprings with potential long‐term effects on their life‐long health.

Human milk oligosaccharides (HMOs) are the third largest nutritional component in breast milk after lactose and fat. 2′‐Fucosyllactose (2′‐FL) is the most abundant HMO in human milk, with a role in promoting *Bifidobacterium* growth and modulating the gut microbiota. HMOs are known to inhibit the growth of harmful bacteria in the intestine of infants, improve the infant's immunity, promote the growth of probiotics in the intestine, and improve the gut microbiome. Currently, the available literature mainly focuses on regulating the composition of intestinal flora, preventing pathogens from adhering to the intestine, immunoregulatory effects, prevention of necrotizing enterocolitis, and promotion of brain development.

Previous studies have reported that supplementing the maternal diet with cholecalciferol, folate, and probiotics during pregnancy can benefit infant development (Basak et al., 2020; Cavalli et al., 2011; Cuinat et al., 2022). However, the effects of including milk oligosaccharides and *Bifidobacterium* lactis *Bb12* as maternal daily diet supplement on the development of the immune system and gut microbiome composition of the offspring remain poorly understood. Herein, the combination of 2′‐FL and *Bifidobacterium Bb12* displayed synergistic interactions that promoted the development of the immune system of the offspring as well as improved their microbiome composition via maternal ingestion. This can be applicable in humans (pregnant women) as well as animals (pregnant females). Therefore, this study aimed to determine the effect of supplementing maternal diet with milk oligosaccharides and *Bifidobacterium Bb12* on the development of gut microbiota and immune response in offsprings.

## 2 MATERIALS AND METHODS

### 2.1 Animals and reagents

A total of 30 female, pregnant Institute of Cancer Research (ICR) mice (age, 8 weeks; weight, 20 ± 2 g; one day following pregnancy) were obtained from Beijing Huafukang Biotechnology Co., Ltd. All procedures in the current study, including animal experiments and sample collection, were approved by the Ethical Committee of Northeast Agricultural University (LA2021276). 2′‐FL (GlyCare™ 2FL 9000) was purchased from Glycom Co., Lid. *Bifidobacterium Bb12* (Probio‐Tec ®BB‐12®Blend‐100IF) was provided by CHR HANSEN Co., Ltd. The cell counting kit‐8 (CCK‐8) were obtained from Sangon Biotech Co., Ltd. The IgG, IgM and IgA ELISA kits were purchased from MultiSciences (Lianke) Biotech Co., Ltd. Takara bacterial genome DNA purification kit was purchased from Takara Co., Ltd. TIANprep rapid mini plasmid kit and fecal DNA purification kit were purchased from Tiangen Biotech (Beijing) Co., Ltd. Lipopolysaccharide was purchased from Biosharp Biotechnology Co., Ltd. The serum‐free RPMI‐1600 medium were obtained from Cienry Co., Ltd. Chicken red blood cells were obtained from Nanjing Maojie Microbial Technology Co., Ltd. 2 × SYBR Green pro TaqHS Premix was purchased from Accurate Biotechnology (Hunan) Co., Ltd.

### 2.2 Experimental design

A total of 30 pregnant mice were randomly divided into six groups (*n* = 5/group). The mice were divided into a control group and five intervention groups, and weighed. From conceive to delivery, each of pregnancy mouse was intragastrically administered the test substance with different concentrations of 2′‐FL and *Bifidobacterium Bb12* that was prepared using 200 μL purified water (Table 1). The control group was fed with 200 μL purified water.

**TABLE 1:** Composition of the test substances used as dietary supplement.

Sample ID^a^	2′‐FL (mg/kg·bw)	*Bifidobacterium Bb12* (cfu/kg·bw)
G_0‐0 (Blank)	0	0
G_1‐0	600	0
G_2‐0	2400	0
G_0‐1	0	2.75 × 10^8^
G_1‐1	600	2.75 × 10^8^
G_2‐1	2400	2.75 × 10^8^

^a^ G_x–y: x stands for 2′‐FLdoses; y stands for *Bifidobacterium Bb12* concentration.

The body weight, eating and drinking water, activity, and defecation of all the female mice were monitored during the intragastric administration of the supplements. Following parturition, the number of mice in each litter, and development of the offsprings were recorded.

The six groups of offspring mice were killed at day 10–12 after birth, and their spleen and blood samples were collected for immune index determination, including immune organ index, splenocyte proliferation, macrophage phagocytosis, Immunoglobulin. The intestinal feces of six offspring mice in each group were collected at day at day 10–12 to detect the content of Lactobacillus and Bifidobacteria in feces of offspring mice.

### 2.3 Determination of immune organ index of the offsprings

The thymus and spleen of nine offspring mice in each group were collected, washed with normal saline, dried using filter paper, and weighed using an electronic balance and their organ indexes were calculated according to the reported formula (Li et al., 2021):

(1) $$\text{Organ}\;\text{index}\;\left(\frac{\mathrm{mg}}{g}\right)=\frac{\text{Organ}\;\text{weight}\;\left(\mathrm{mg}\right)}{\text{Body}\;\text{weight}\;\left(g\right)}$$

### 2.4 Lipopolysaccharide (LPS)‐induced splenocyte proliferation experiment

Splenocyte proliferation was evaluated using CCK‐8 assay (Wu & Huang, 2021). The mouse spleens were placed in a small dish containing sterile Hank's solution. Tweezers were used to gently shred the spleen into a single‐cell suspension, which was then filtered through a 200‐mesh sieve, washed, and the cell concentration was adjusted to 2.5 × 10^6^ cells/mL with phosphate buffered saline i.e., PBS. 96‐well culture plates were seeded with 2.5 × 10^5^ cells per well in100 μL and then 100 μL LPS (at a final concentration of 10 μg/mL) were added to the wells. Furthermore, serum‐free RPMI‐1600 medium (containing 100 U/mL penicillin and 100 μg/mL streptomycin) was used as the control group. After 72‐h incubation, 10 μL of CCK‐8 solution was added to each well and incubation was performed for another 1 h. Then, the optical density value was measured at 490 nm using a spectrophotometer. The splenocyte proliferation ratio (%) was calculated as follows:

(2) $$\begin{array}{c} \text{Splenocyte}\;\text{proliferation}\;\text{ratio}\;\left(\%\right)\\ =\frac{\mathrm{OD}\;\text{value}\;\left(\mathrm{LPS}\right)-\mathrm{OD}\;\text{value}\;\left(\text{normal}\right)}{\mathrm{OD}\;\text{value}\;\left(\text{normal}\right)}\times 100\% \end{array}$$

### 2.5 Macrophage phagocytosis

Six 10‐day‐old offspring mice in each group were intraperitoneally injected with 0.8 mL of 5% sterile starch solution. After 3 days, the mice were intraperitoneally injected with 0.8 mL of 1% chicken red blood cells (CRBC). After 30 min, the mice were euthanized via cervical dislocation and 2 mL of saline was intraperitoneally injected; then, 1 mL of peritoneal fluid was aspirated after 1 min and smeared on two glass slides followed by incubation at 37°C for 30 min. After complete drying, the smears were stained with Wright's dye for 5 min, dried and sealed with neutral resin, and the number of macrophages were counted under an oil immersion lens of a compound microscope (Liu et al., 2023). The percentage of phagocytosis was analyzed using the following formula:

(3) $$\begin{array}{c} \text{Phagocytosis}\;\text{percentage}\;\left(\%\right)\\ =\frac{\text{Number}\;\text{of}\;\text{macrophages}\;\text{that}\;\mathrm{had}\;\text{engulfed}\;\text{CRBC}}{\text{Number}\;\text{of}\;\text{macrophages}}\times 100\% \end{array}$$

### 2.6 Determination of immunoglobulin in serum

Six offspring from each group were anesthetized by intraperitoneal injection with sodium pentobarbital, and decapitate. Their blood samples were centrifuged at 1000 *g* at 4°C for 10 min. Then, the supernatants were collected to obtain the plasma and stored at −80°C. The immunoglobulin G (IgG), immunoglobulin (IgA), and immunoglobulin M (IgM) in the plasma were determined using enzyme‐linked immunosorbent assay kit according to the manufacturer's instructions.

### 2.7 Conventional polymerase chain reaction, sequencing, and cloning

*Lactobacillus* and *Bifidobacterium* DNA was extracted using Takara bacterial genome DNA purification kit according to the manufacturer's instructions. Conventional polymerase chain reaction (PCR) was conducted using the previously developed primers (Table 2) targeting *La2‐1* of *Lactobacillus* and *BIF‐2* of *Bifidobacterium*. The PCR products were subsequently subjected to bidirectional sequencing and compared with known sequences in GenBank using TBLASTX application or software. For cloning, the PCR products from La2‐1 and BIF‐2 were cloned into *Escherichia coli* (Takara, China). The cloned products were verified via bidirectional sequencing. The purification and linearization of plasmids were performed according to the manufacturer's instructions using TIANprep rapid mini plasmid kit and quantified via spectrophotometry.

**TABLE 2:** Primers used in quantitative real‐time PCR assay and amplification of fragments of La2‐1 and BIF‐2 genes by standard PCR for cloning.

Target gene	Primer sequence	Amplicon (bp)
La2‐1 (*Lactobacillus*)	F: 5′‐CGCAAGTGGCTTGCTTAGTC‐3′	224
	R: 5′‐CCACTGTCCGTACCAGCAAT‐3′	
BIF‐2 (*Bifidobacterium*)	F: 5′‐CATCCGGCATTACCACCC‐3′	511
	R: 5′‐CCACCGTTACACCGGGAA‐3′	

### 2.8 Construction of real‐time quantitative polymerase chain reaction standard curves

The cloned plasmids were subjected to a 10‐fold gradient dilution, with 45 μL of ddH_2_O and 5 μL of the plasmid. Assays were performed in triplicate on dilutions of a turtle‐derived positive control plasmid of the La2‐1 gene of *Lactobacillus* and the BIF‐2 gene of *Bifidobacterium* within a single run. The standard PCR reaction mixture (10 μL) was used, with the following reagents added in sequence: 2 × SYBR Green pro TaqHS Premix (5.0 μL), forward and reverse primers (0.4 μL; Table 2), ROX reagent (0.2 μL), DNA template (1.0 μL), and DNase‐ and RNase‐free water (3.0 μL). The reaction was performed under the following conditions: pre‐denaturation at 95°C for 60 s, 40 cycles of denaturation at 95°C for 10 s, annealing at 60°C for 30 s, and extension at 72°C for 30 s, followed by a final extension at 72°C for 10 min to collect the signal. Standard curves were generated using the cycle threshold values of the positive control plasmid dilutions.

### 2.9 Detection of *Bifidobacterium* and *Lactobacillus* in feces of offspring via real‐time polymerase chain reaction

The intestinal feces of six offspring mice in each group were collected and put into an aseptic Eppendorf (EP) tube and weighed. The genomic DNA of all the bacteria in feces was extracted using a fecal DNA purification kit and stored at −80°C. According to the standard reaction system and reaction conditions, the DNA extract obtained from the feces of offspring mice was reacted via real‐time fluorescence quantitative PCR. The cycle threshold (Ct) values of each sample were normalized (brought into the standard curve), and the copy numbers of *Lactobacillus* and *Bifidobacterium* genes per microgram DNA were calculated.

### 2.10 Statistical analysis

All data are presented as mean ± standard deviation. The experimental results were subjected to analysis of variance (ANOVA) using SPSS (15.0) software. The homogeneity of variance in one‐way ANOVA was conducted using least significant difference test, and nonparametric rank sum test or two‐sample independent *T*‐test was used for uneven variance. Figures were generated using GraphPad Prism 8.0 software. *p* < .05 was considered statistically significant.

## 3 RESULTS

The female mice ate a normal diet and drank water throughout the pregnancy, and their weight increased gradually in the early period and rapidly in the later period. None of the offspring showed any deformity after birth and showed good growth.

### 3.1 The spleen and thymus gland index of offspring mice

**FIGURE 1:**
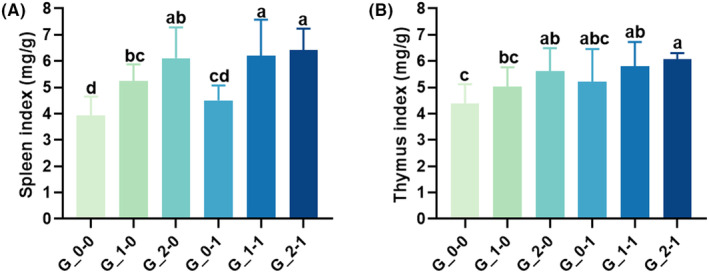
(A) Effect of maternal diet supplementation with milk oligosaccharides and probiotics on the spleen index of offspring mice. (B) Effect of maternal diet supplementation with milk oligosaccharides and probiotics on the Thymus index of offspring mice. Different letters indicate significant differences among the groups (*p* < .05).

As shown in Figure 1, after ICR pregnant mice were treated with different doses of 2′‐FL, the spleen and thymus indexes of offspring were significantly higher than those of the blank control group (*p* < .05), and the spleen and thymus indexes of offspring under the influence of *Bifidobacterium Bb12* were higher than those of the blank control group, but the difference was not significant (*p* > .05). When ICR pregnant mice were treated with 2′‐FL and *Bifidobacterium Bb12* simultaneously, the immune organ indexes of the offspring mice were significantly higher than those of the single‐factor intervention groups (*p* < .05). This shows that the combination of 2′‐FL and *Bifidobacterium Bb12* can promote the growth of immune organs of offspring mice, increase the indexes of immune organs, and promote the development of the immune system.

### 3.2 Serum immunoglobulin content (IgG, IgA, and IgM) of offspring mice

**FIGURE 2:**
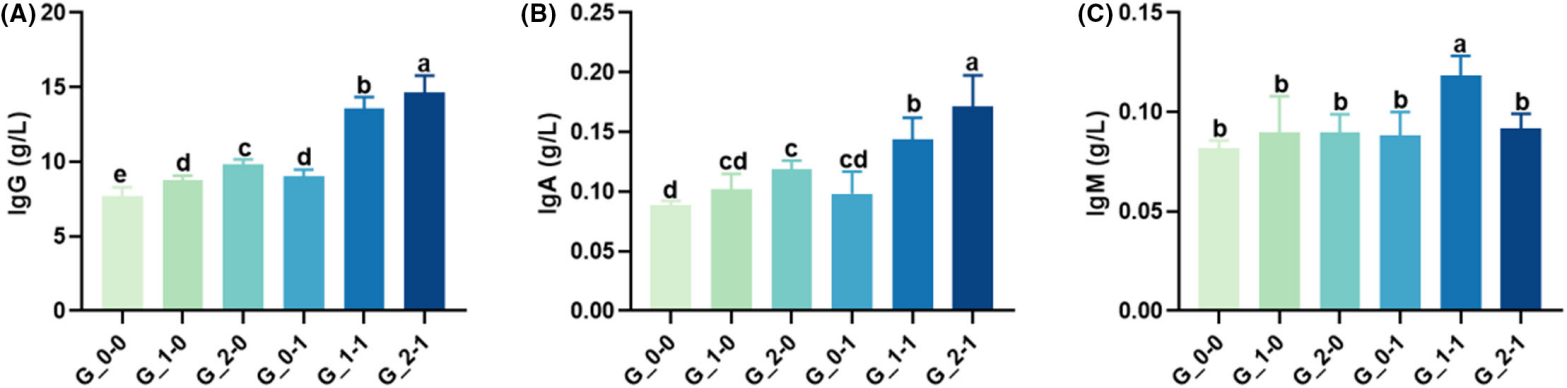
Effect of maternal diet supplementation with milk oligosaccharides and probiotics on the serum IgG (A), IgA (B), and IgM (C) concentration in offspring mice. Different letters indicate significant differences among the groups (*p* < .05).

After the ICR pregnant mice were treated with different doses of 2′‐FL, the serum IgG content of offspring mice significantly increased compared with the blank control group (*p* < .05), and the increase was dose dependent (Figure 2A). Under the intervention of *Bifidobacterium Bb12*, the serum IgG content of offspring mice significantly increased compared with the blank control group (*p* < .05). Compared with the single‐factor intervention group and the control group, the combination of 2′‐FL and *Bifidobacterium Bb12* exhibited a significant increase in the content of IgG in the serum of offspring mice (*p* < .05). Figure 2B,C represent the levels of IgA and IgM, respectively. When compared with the blank control group, the serum IgA content of offspring mice increased following 2′‐FL intervention. However, the difference was only in the higher concentration group (G_2‐0); there was a significant difference in the IgM content (*p* < .05). The IgM content remained unaffected except for in G_1‐1. No significant change was observed in the serum IgA and IgM contents of the offspring mice under *Bifidobacterium Bb12* intervention compared with the blank control group. However, compared with the single‐factor intervention and control groups, the combination of 2′‐FL and *Bifidobacterium Bb12* significantly increased the serum IgA and IgM content in offspring mice.

### 3.3 Spleen lymphocytes of offspring mice

**FIGURE 3:**
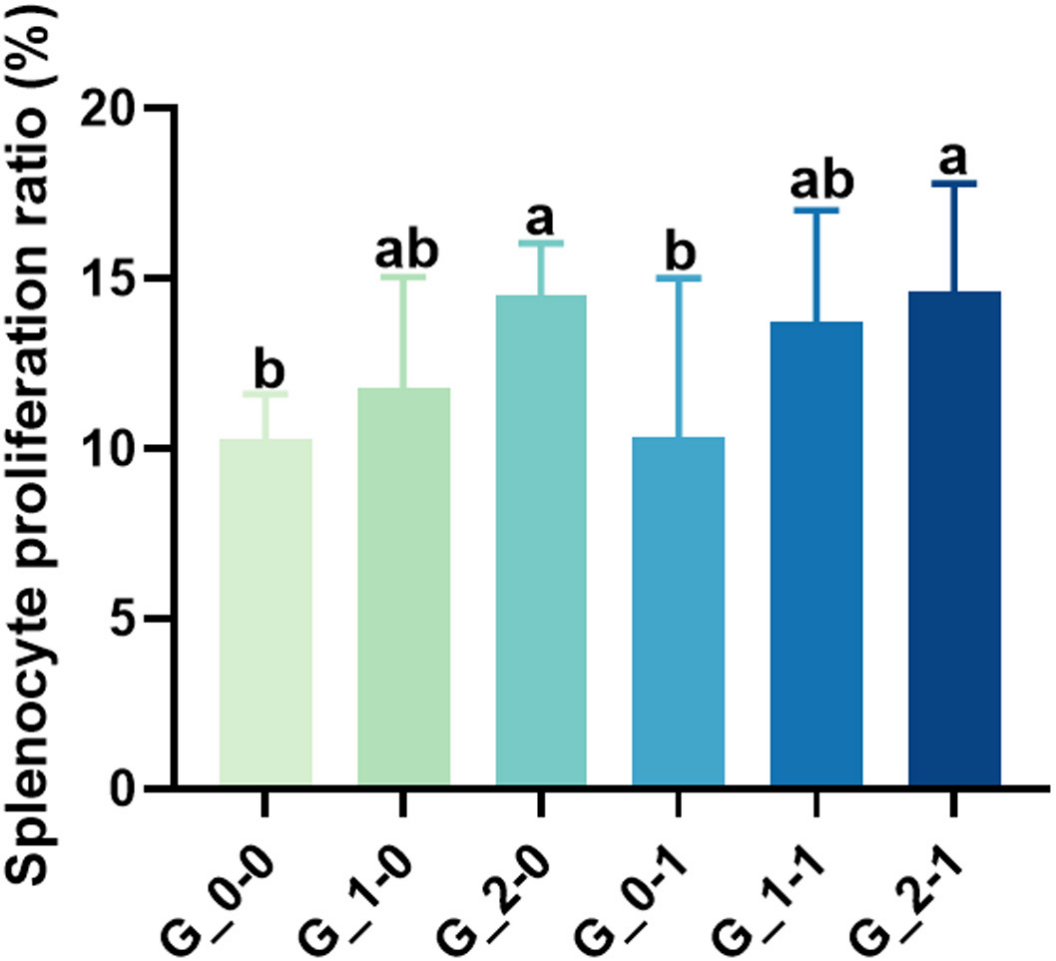
Effect of maternal diet supplementation with milk oligosaccharides and probiotics on the spleen lymphocytes of offspring mice. Different letters indicate significant differences among the groups (*p* < .05).

Figure 3 shows that following the treatment of ICR pregnant mice with different doses of 2′‐FL, the proliferation percentage of spleen lymphocytes of the G_1‐0 group increased after 72 h of LPS induction; however, the difference was not significant compared with the blank control group. The G_2‐0 group showed a significantly higher percentage of spleen lymphocyte proliferation. Intervention with *Bifidobacterium Bb12* did not show a significant change after 72 h of LPS induction (*p* > .05) compared with the blank control. However, when the combination of 2′‐FL and *Bifidobacterium Bb12* was administered, the spleen lymphocyte proliferation percentage in offspring mice after 72 h of LPS induction was significantly higher than that of the blank control group. However, there was no significant change compared with the intervention of 2′‐FL alone.

### 3.4 The phagocytic activity of macrophages in offspring mice

**FIGURE 4:**
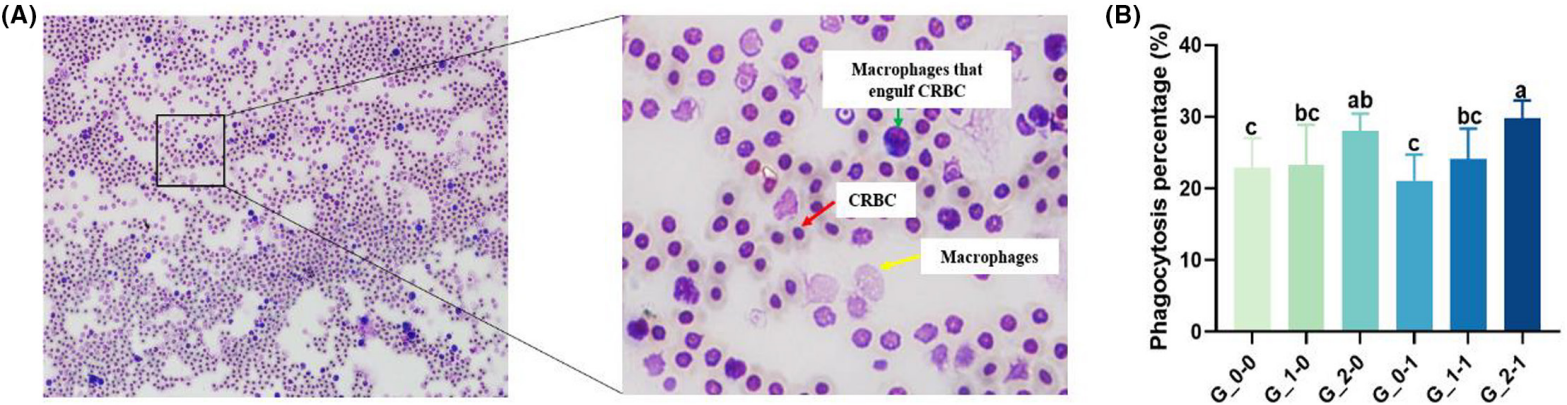
(A) Phagocytic activity of offspring mice macrophages against CRBC (Macrophage phagocytic chicken red blood cell) representative image. (B) Effect of maternal diet supplementation with milk oligosaccharides and probiotics on the phagocytic activity of macrophages in offspring mice. Different letters indicate significant differences among the groups (*p* < .05).

As depicted by Figure 4B, 2′‐FL intervention resulted in the higher percentage of macrophage phagocytosis in the offspring as compared to the blank control group. The difference is not significant between G_0‐0 group and G_1‐0 group (*p* > .05), but the difference is significant between the G_0‐0 group and the G_2‐0 group (*p* < .05). *Bifidobacterium Bb12* intervention showed no significant difference in the percentage of macrophage phagocytosis of the offspring as compared with the blank control group (*p* > .05). When the combination of 2′‐FL and *Bifidobacterium Bb12* was used, the phagocytosis percentage of macrophages in offspring mice was significantly higher than that in the blank control group (*p* < .05). However, there was no significant difference between the G_1‐0 group and the G_1‐1 group (*p* > .05), the same as the G_2‐0 group and the G_2‐1 group (*p* > .05).

### 3.5 Occurrence of probiotics in the feces of offspring mice

**FIGURE 5:**
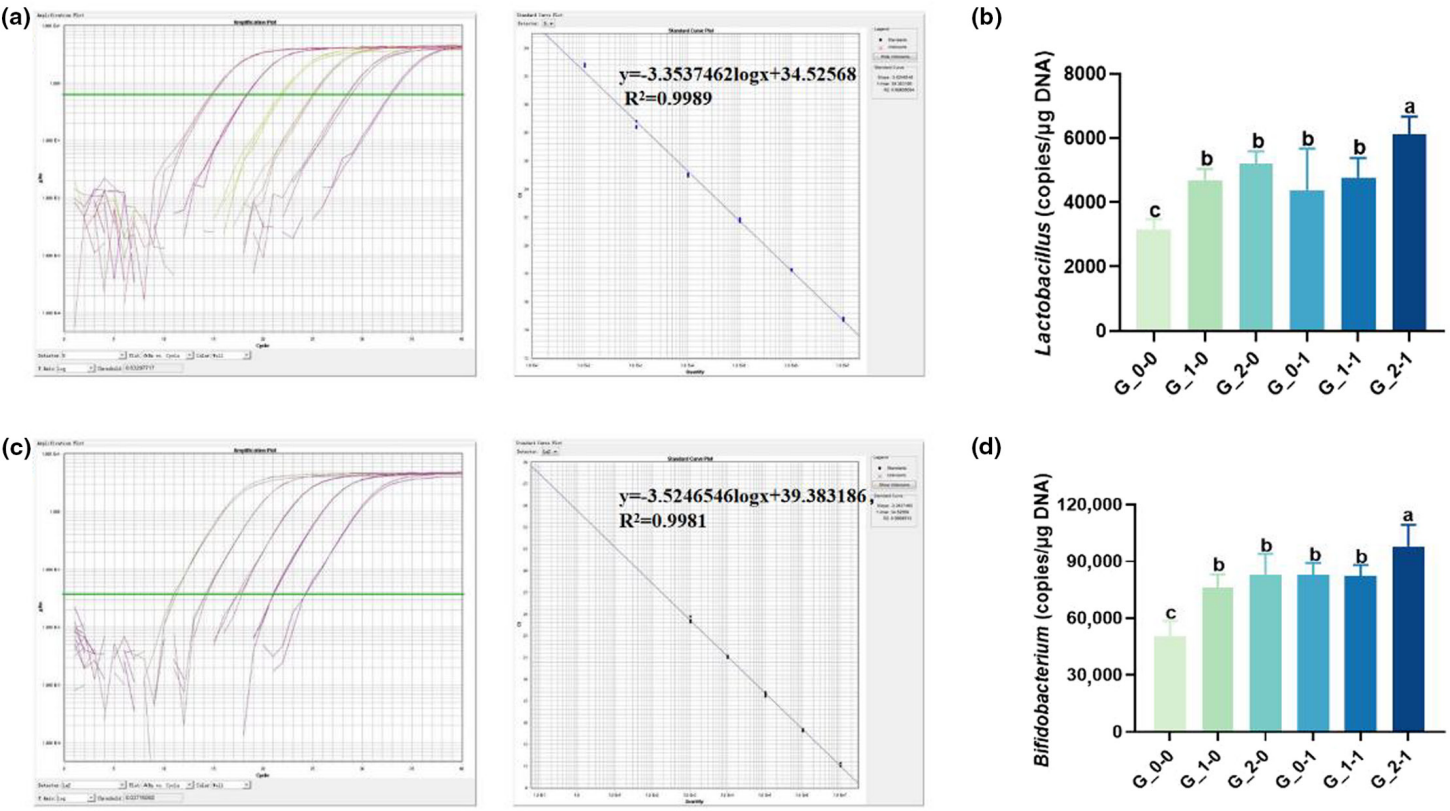
(A) The qPCR amplification curves and standard curves of *Lactobacillus* plasmids. (B) Effect of maternal diet supplementation with milk oligosaccharides and probiotics on the *Lactobacillus* in feces of offspring mice. (C) The qPCR amplification curves and standard curves of *Bifidobacterium* plasmids. (D) Effect of maternal diet supplementation with milk oligosaccharides and probiotics on the *Bifidobacterium* in feces of offspring mice. Different letters indicate significant differences among the groups (*p* < .05).

Figure 5 shows that when ICR pregnant mice were treated with different doses of 2′‐FL, the abundance of *Lactobacillus* and *Bifidobacterium* in the feces of offspring mice was significantly higher compared with the blank control group (*p* < .05), indicating that the 2′‐FL intervention in maternal diet improved the intestinal flora of the offspring. An almost identical trend was observed with the *Bifidobacterium Bb12* intervention wherein the abundance of *Lactobacillus* and *Bifidobacterium* in the feces of offspring mice was significantly higher than that in the blank control group (*p* < .05), indicating the potential of *Bifidobacterium Bb12* intervention to improve the intestinal flora of offspring to a certain extent. The abundance of probiotics in the feces of offspring mice remained unchanged in the single‐factor intervention group when low concentrations of 2′‐FL and *Bifidobacterium Bb12* were used (*p* > .05). However, the abundance significantly increased when a high concentration of 2′‐FL and *Bifidobacterium Bb12* was supplemented in combination in the single‐factor intervention group (*p* < .05).

## 4 DISCUSSION

The intestinal microbiome of humans or animals has a very important function in the development of intestinal immune system and the intestinal mucosal barrier function (Dzidic et al., 2018; Gao et al., 2018). Studies have shown that probiotics can regulate the intestinal barrier function and activate mucosal immune responses (George Kerry et al., 2018; Rodríguez‐Viso et al., 2023). As an ideal human or animal dietary supplement, prebiotics can promote the growth and metabolism of probiotics and further enhance the efficacy of probiotics (Yang et al., 2019). The combined use of probiotics and prebiotics is already a well‐known strategy. However, the effects of the maternal dietary supplement of probiotics and prebiotics on the offspring gut microbiota, especially on the immune function of the offspring, are rarely studied. There are very few reports available regarding this and the conclusions are not uniform (George Kerry et al., 2018; Rodríguez‐Viso et al., 2023).

Breast milk is the most natural and nutritious food source for infants and young children (Kullmann et al., 2022). The HMOs in breast milk can be used by intestinal microorganisms to selectively stimulate the growth of beneficial intestinal bacteria, such as *Bifidobacterium* spp. and *Lactobacillus* spp., which positively impact the formation of the intestinal immune barrier (Seppo et al., 2019).

Immune organs are directly responsible for the immunity of the body. The spleen and thymus are important immune centers of the body were T and B cell differentiation, development, and maturity occurs. They also serve as the center for cell‐mediated and humoral immunity. Therefore, the spleen and thymus indexes can reflect the body's immune capacity to a reasonable extent (Tang et al., 2022). T and B lymphocytes generate in the spleen, and the proliferation capacity of lymphocytes reflects the immune strength of the body (Attia et al., 2017). Reportedly, HMOs contribute to the maturation of lymphocytes and maintain the balance between the production of T helper cells (Th) 1 and Th 2 cytokines (Eiwegger et al., 2004). Herein, when the maternal mice were fed 2′‐FL, the spleen and thymus indexes of the offspring mice significantly increased, whereas these indexes did not significantly change in the offspring mice after intervention with *Bifidobacterium Bb12* alone. Similarly, supplementing with 2′‐FL alone significantly improved the lymphocyte proliferation capacity of the offspring mice, whereas the addition of *Bifidobacterium Bb12* alone exerted little effect on the lymphocyte proliferation ability of offspring mice. When 2′‐FL was combined with *Bifidobacterium Bb12*, after LPS induction for 72 h, there was no significant change in the proliferation rate of splenic lymphocytes in the offspring mice compared with that of the offspring mice treated with the same concentration of 2′‐FL alone. Therefore, we speculate that the maternal administration of 2′‐FL plays a dominant role in the development of the immune organs of the offspring and lymphocyte proliferation.

Reportedly, each *Bifidobacterium* living in the infant gut and bacteria of other genera use distinct molecular mechanisms to capture and digest differentially structured HMOs to avoid competition, thereby successfully maintaining the dynamic balance of the gut (Salli et al., 2020). In previous studies, the effect of breast milk oligosaccharides on the regulation of gut microbiota was inconsistent (Hu et al., 2023; Wang, Hu, et al., 2021; Wang, Ze, et al., 2021; Zhang et al., 2021). Herein, intervention with 2′‐FL alone in pregnant mice significantly increased the abundance of *Lactobacillus* and *Bifidobacteria* in the feces of the offspring mice, with the addition of *Bifidobacterium Bb12* alone also exhibiting a similar effect. Notably, with the simultaneous addition of *Bifidobacterium Bb12* and 2′‐FL (G_2‐1), the abundance of *Bifidobacteria* in the progeny feces was significantly higher than the other groups. Similarly, with the simultaneous addition of *Bifidobacterium Bb12* and 2′‐FL (G_2‐1), the abundance of *Lactobacillus* in the progeny feces was significantly higher than the other groups. This indicates that maternal diet supplementation with 2′‐FL or *Bifidobacterium Bb12* improves the abundance of beneficial bacteria in the intestines of the offspring mice. Additionally, the combination of 2′‐FL and *Bifidobacterium Bb12* was observed exhibit a better performance that either of them alone. We speculated that the possible reason was that maternal supplementation of probiotics or prebiotics could significantly change the content of probiotics in the intestinal tract and around the skin tissue of maternal mice. Some probiotics were also distributed in the amniotic fluid, and probiotics were obtained in the process of swallowing amniotic fluid or sucking breast milk, thus increasing the abundance of bacteria in the intestinal tract.

IgM in the blood is the earliest antibody appearing in the process of an immune response, which has the functions of binding complement and dissolving pathogens (Wang et al., 2022). IgA is second only to IgG in serum and has significant effects on improvement of immunity against pathogens introduced through the mucosal pathway. In this study, the maternal diet supplementation with 2′‐FL or *Bifidobacterium Bb12* alone had little effect on IgM and IgA contents in offspring mice, particularly the addition of *Bifidobacterium Bb12* alone, had no effect on IgA and IgM in offspring mice, suggesting that *Bifidobacterium Bb12* supplementation hardly affects the intestinal IgA contents. This is similar to the previous study (Taheri et al., 2022). IgG is believed to be the only maternal antibody capable of crossing the placental barrier, and IgG secreted by the mammary glands enters the colostrum, enabling the newborn to be protected by the maternal antibodies for the first time (Ben Suleiman et al., 2012). In this study, the addition of 2′‐FL or *Bifidobacterium Bb12* alone promoted the IgG content in the serum of offspring mice. When the two were used together, the IgG content in the serum of offspring mice was found to increase significantly. A supplement of 2′‐FL or *Bifidobacterium Bb12* increases autoantibody levels, and IgG crosses the placental barrier into the colostrum, thus raising IgG content in the serum of offspring mice, which is more effective when combined. We speculated that the maternal mice supplemented with probiotics and prebiotics increased the immunoglobulin level in breast milk, and the offspring mice increased their serum immunoglobulin content through breastfeeding.

In terms of nonspecific immunity, mononuclear macrophages are an important executor of nonspecific immunity, which is responsible for the phagocytosis of pathogenic microorganisms, the removal of damaged cells, and the uptake of antigens, and the stimulation of immune response (Castro‐Alves & Nascimento, 2021). Macrophages play an important role in the organism's immune response by secreting pro‐inflammatory cytokines, such as carbon monoxide, signaling proteins, and several other inflammatory mediators (Muniandy et al., 2018). Previous studies have shown that prebiotics, such as fructooligosacharide and inulin (Vogt et al., 2013), mannan oligosaccharide (MOS; Ferenczi et al., 2016), neoagarooligosaccharides (NAOS; Wang et al., 2017). And oligosaccharides from dragon fruit are capable of directly modulating the immune system including antiinflammatory responses (Nattha et al., 2021). Herein, the ability of offspring mice macrophages to engulf chicken erythrocytes was significantly enhanced by the 2′‐FL intervention, indicating that the administration of 2′‐FL by the maternal mice could promote the phagocytosis of offspring macrophages. Previous results demonstrated that prebiotics can modulate the alternate activation of macrophages and contribute to reshaping mucosal immunity in infants and young children. The supplementation of *Bifidobacterium Bb12* alone did not affect the phagocytosis by offspring mice macrophages, when 2′‐FL and *Bifidobacterium Bb12* were used in combination, phagocytosis of offspring mouse macrophages could be further enhanced.

## 5 CONCLUSION

When maternal mice were supplemented with 2′‐FL alone, the immunity level of the offspring mice increased, gut microbiota improved, and abundance of beneficial bacteria increased and the improvement was dose dependent. When supplemented with *Bifidobacterium Bb12* alone, there was little effect on the immunity level of the offspring mice; however, the abundance of beneficial bacteria in the intestine of the offspring mice significantly increased. When 2′‐FL and *Bifidobacterium Bb12* were administered in combination, the spleen and thymus indexes, serum immunoglobulin content, macrophage phagocytic capacity, and intestinal abundance of beneficial bacteria were significantly higher in the offspring mice than in the offsprings of female mice who were fed with supplement alone. Therefore, the combined use of 2′‐FL and *Bifidobacterium Bb12* exhibited better improved the immunity and intestinal flora of offspring mice compared with the use of either of them alone. Overall, 2′‐FL and *Bifidobacterium Bb12* supplementation may be a safe method for regulating the establisment of the intestinal flora and acquired immune system development. However, it is not clear how maternal microbes affect fetal immune mechanisms and whether it can be applied to human immune regulation., which should be explored in greater depth.

## AUTHOR CONTRIBUTIONS

**Qinggang Xie:** Conceptualization (equal); funding acquisition (equal); investigation (equal); methodology (equal); writing – review and editing (equal). **Dongying Cui:** Data curation (equal); formal analysis (equal); investigation (equal); methodology (equal); supervision (equal). **Qinchao Zhu:** Investigation (equal); methodology (equal); writing – original draft (equal). **Xuewen Qin:** Investigation (equal); methodology (equal); writing – original draft (equal). **Daxi Ren:** Conceptualization (equal); methodology (equal); project administration (equal); supervision (equal); writing – review and editing (equal). **Xiaoxi Xu:** Conceptualization (equal); funding acquisition (equal); project administration (equal); resources (equal); writing – review and editing (equal).

## CONFLICT OF INTEREST STATEMENT

The authors declared that they have no conflict of interest to this work.

## ETHICAL APPROVAL

The study was approved by the Ethical Committee of Northeast Agricultural University (LA2021276).

## Data Availability

Data are available on reasonable request from the corresponding author.
